# A poor prognostic case of peripheral T-cell lymphoma in the base of tongue with chemotherapy followed by radiation therapy

**DOI:** 10.1186/2193-1801-3-731

**Published:** 2014-12-13

**Authors:** Jun-Ho Lee, Seok Ho Lee

**Affiliations:** Department of Emergency Medical Technology, Daejeon University, 62 Daehak-ro, Dong-gu, Daejeon, 300-716 Korea; Department of Radiation Oncology, Gil Medical Center, School of Medicine, Gachon University, 1198 Guwol-dong, Namdong-gu, Incheon, 405-760 Korea

**Keywords:** Base of tongue, Peripheral T cell lymphoma, Radiation Therapy

## Abstract

A 59-year old male had odynophagia and globus sensation for 10 days prior to his visit. Endoscopically, a fungating mass was observed in the right side of the tongue base (BOT). The peripheral T-cell lymphoma (PTCL) was confirmed by a punch biopsy. The patient was classified as stage IIA according to the Ann Arbor staging classification. Radiation therapy (RT) using volumetric modulated arc therapy (VAMT) began after 8 cycles of CHOP chemotherapy. Although the patient achieved a complete remission without RT relating morbidity during the follow-up period, recurrence occurred in the right orbit at 3 months after treatment. The patient received RT with high energy electron on the right orbit. The salvage chemotherapy was followed by RT. The patient died of aggravation of PTCL at 17 months after VMAT. We report a poor prognostic case of PTCL in BOT with chemotherapy followed by RT.

**Electronic supplementary material:**

The online version of this article (doi:10.1186/2193-1801-3-731) contains supplementary material, which is available to authorized users.

## Background

The incidence of Non-Hodgkin’s lymphoma (NHL) of the oral cavity accounts for 3-5% of all malignant lesions of the oral cavity. Further, only 1% of all lymphomas are primary oral cavity lymphoma. NHL in the base of tongue (BOT) is even rarer (Lee et al.^1987^); (Zapater et al.^2010^). Primary oral cavity lymphoma occurs in Waldeyer`s ring, including the tonsils, nasopharyngeal lymphoid tissue, soft palate, and BOT (Lee et al.^2014^). Diffuse large B cell lymphoma (DLBCL) has been reported as the most common type of primary oral NHL (Kemp et al.^2008^). However, the Peripheral T-cell lymphomas (PTCL) in the head and neck (H&N) region were rarely reported (Jaffe^2002^). Because of its rarity, the treatment guidelines for primary BOT lymphoma have not been clearly established (Guastafierro et al.^2008^). Generally, the standard treatment for patients with early stage DLBCL is chemotherapy followed by involved field radiation therapy (IFRT) (You et al.^2004^). Among H&N lymphoma cases treated with IFRT followed by chemotherapy, most were traditionally treated with three dimensional conformal radiation therapy (3D-CRT) (Chang et al.^2009^). It has been well known that 3D-CRT in H&N cancer patients could increase the incidence of xerostomia, whereas the volumetric modulated arc therapy (VMAT) could significantly reduce the incidence of RT-induced toxicity (Holt et al.^2013^). We here report a poor prognostic case of PTCL in BOT with chemotherapy followed by RT with the use of VMAT.

## Case description

**Figure 1:**
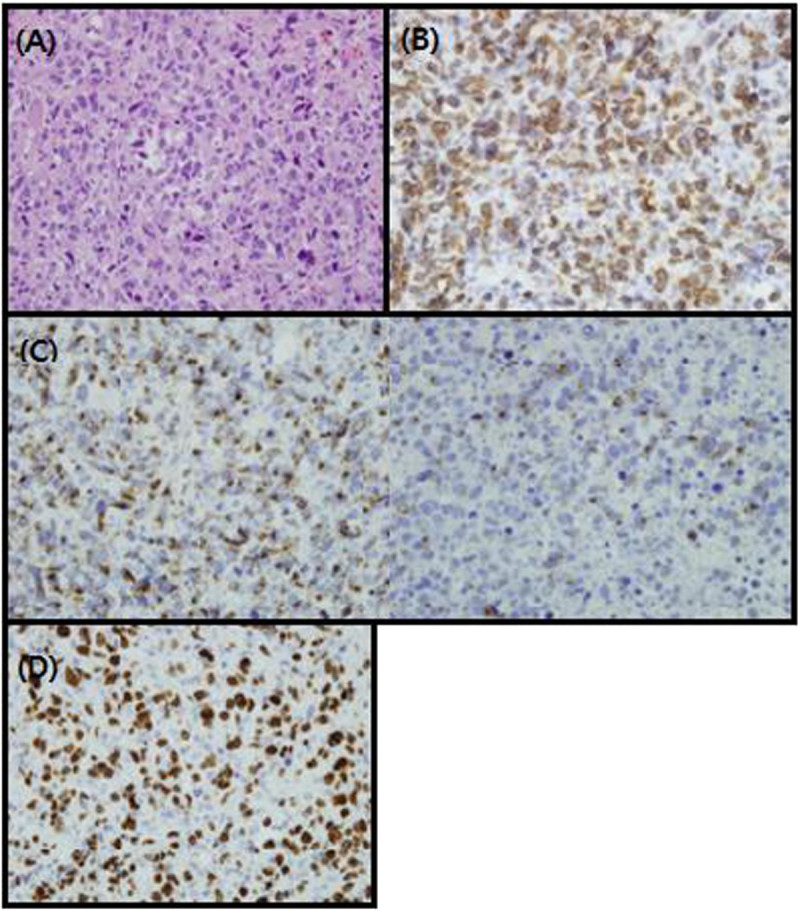
**A punch biopsy of the tongue base showed a diffuse dense infiltration of the lymphoid cells (x 400). (A)** Immunohistochemical staining of tumor cells. **(B)** The tumor cells showed a diffuse cytoplasmic immunopositivity for CD3 (x400), but tumor cells were immunonegative for CD20, CD30, and CD56. The immunophenotype of tumor cells were mixed pattern for CD4 and CD8. **(C)** The tumor cells expressed cytotoxic granule associated proteins TIA-1 and granzyme B. **(D)** The proliferation index by Ki-67 immunostain expressed with a high level (about 80%).

A 59-year old male had odynophagia and globus sensation for 10 days before his visit. A fungating mass was observed endoscopically on the right side of the tongue base. The tumor mass originated from the base of the tongue and extended to the right pyriform sinus. The tumor measured approximately 3 cm longitudinally and 2.7 cm transversally. The patient experienced no B symptoms (fever, weight loss, and night sweats). The Eastern Cooperative Oncology Group (ECOG) performance status of the patient was 0–1. The laboratory results at the time of admission were as follows: lactate dehydrogenase (LDH), 380 U/l) (normal range, 120–520); white cell count, 6,040/μl (normal range, 4,000-10,000); hemoglobin, 14.0 g/dl (normal range, 13–17); platelet count, 289,000/μl (normal range, 150–450); aspirate aminotransferase, 26 IU/l (normal range, 0–40); alanine transaminas, 21 IU/l (normal range, 5–40); blood urea nitrogen, 16.7 mg/dl (normal range, 8–22); and creatinine, 0.7 mg/dl (normal range, 0.6-1.2). Pathologic observation suggested T-lineage lymphoid malignancy. The immunohistochemistry panel used to define the diagnosis was as follows: a panel of monoclonal antibodies against CD 3, CD 4, CD 8, CD 20, CD 30, CD 56, TIA-1, Granzyme B (all from DAKO, Copenhagen, Denmark), and Ki-67(DAKO, Glostrup, Denmark). Using ISH (in situ hybridization) technique, Epstein-Barr virus(EBV)-encoded RNA (EBERs) was detected. Paraffin sections were pretreated with xylene followed by proteinase K (Merck, Darmstadt, Germany), treatment, which was hybridized with fluorescein isothiocyanate-conjugated EBV oligonucleotides (Novocastra, Newcastle, U.K.) complementary to the mRNA portion of the EBER genes. A confirmative diagnosis of punch biopsy was PTCL, not otherwise specified (NOS) (Figure 1).

**Figure 2:**
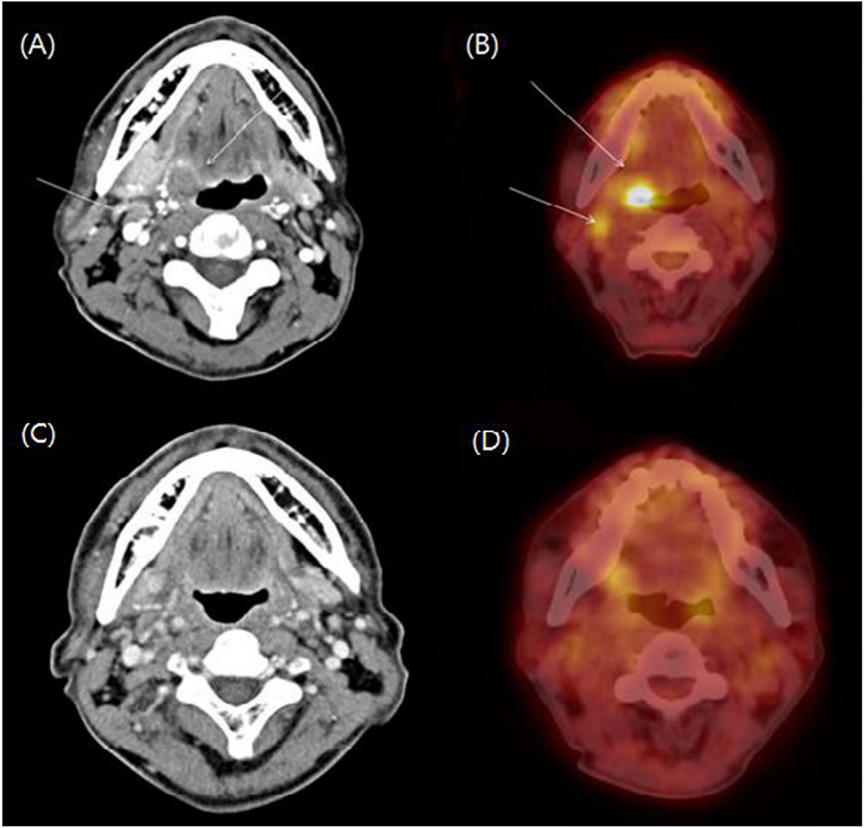
**F-18 FDG PET and CT scan findings. (A)** Ill defined heterogeneous enhancing soft tissue mass lesion in the right tongue base and enlarged lymph nodes in the right level II are observed on a plain contrast CT scan. **(B)** F-18 FDG PET reveals a hypermetabolic lesion (arrow) in the right tongue base and lymph node in the right level II. **(C)** A follow-up CT scan reveals no evidence of mass lesion in the right tongue and no significant lymph node in both necks after the treatment. **(D)** After the treatment, a 1 month follow-up PET scan shows disappearance of the previous hypermetabolic lesion.

Tumor cells were immunonegative for EBERs and Human T-cell leukemia virus type 1. The staging work-up was performed according to the Ann Arbor staging classification. A computed tomography (CT) scan of the H&N and positive emission tomography (PET) were evaluated. A bone-marrow (BM) biopsy was also performed. H&N CT showed an ill-defined heterogeneous enhancing soft tissue mass in the right tongue base, which extended to the right pyriform sinus. There were slightly enlarged enhancing lymph nodes in the right neck at level II. F-18 FDG PET revealed a hypermetabolic lesion (SUV max 8.18) in the right tongue base and lymph node (SUV max 1.5) in the right neck level II (Figure 2). BM biopsy showed a negative result for lymphomatous infiltration. Lumbar puncture was not attempted in this patient.

**Figure 3:**
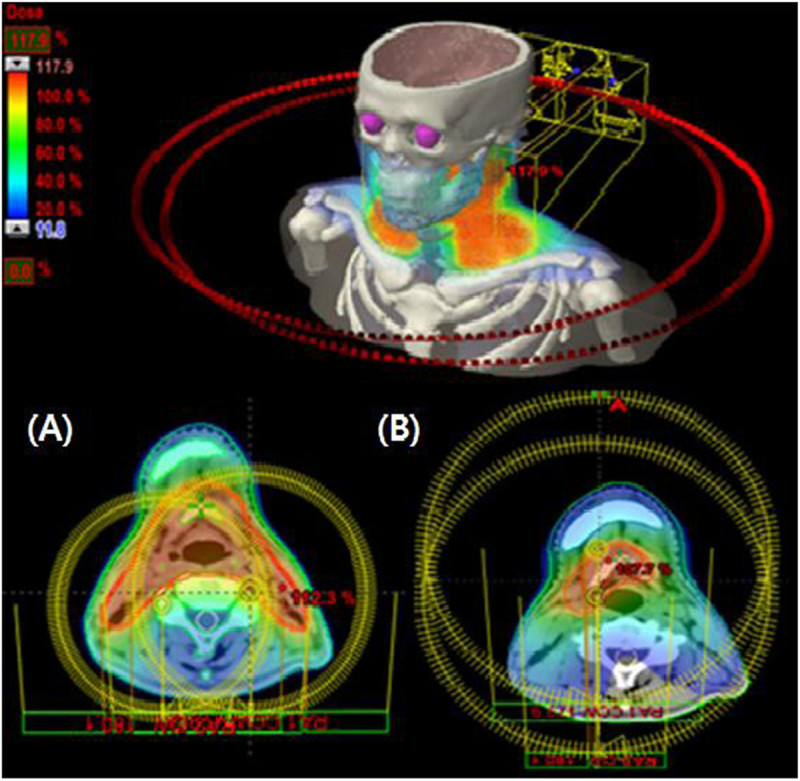
**The patient received volumetric modulated arc therapy for parotid gland sparing with (A) dose prescription of 3,450 cGy in 15 fractions over 3 weeks plus (B) a local boost of 1,610 cGy to the primary site in 7 fractions over 1 week.** The total RT dose was delivered 50.6 Gy in 22 fractions over 4.5 weeks.

**Figure 4:**
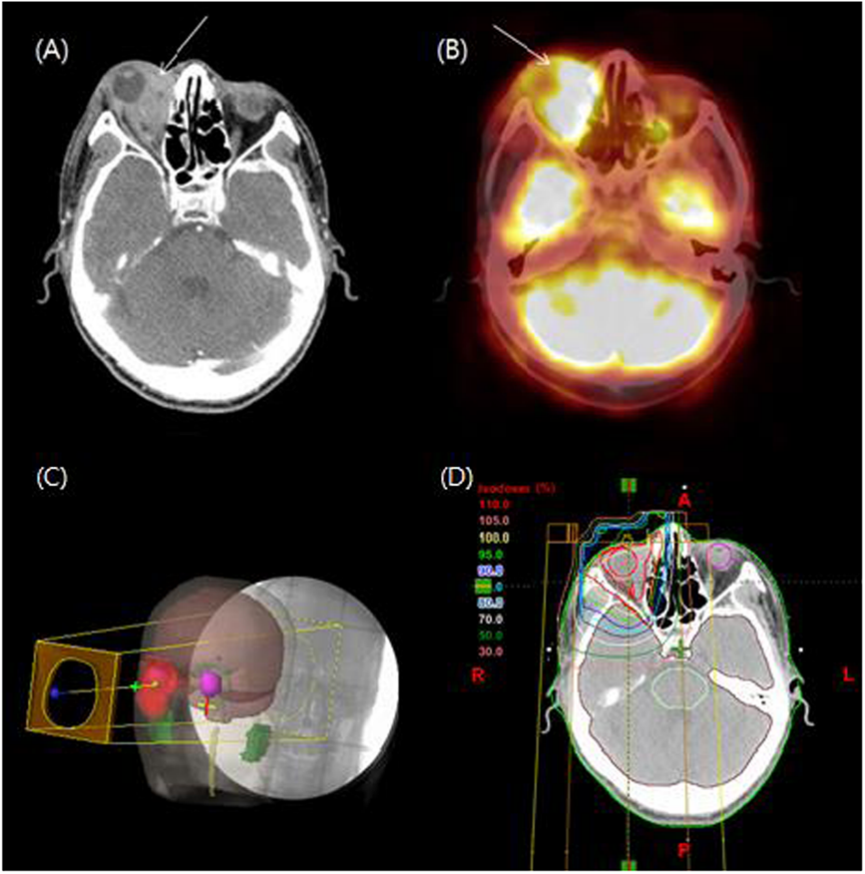
**Compared to CT and PET scan 3 months ago. (A)** Follow-up CT scan shows developed homogenous enhancing lesion surrounding the right orbit and right lower eyelid, and **(B)** the hypermetabolic lesion in the right orbit were newly disappeared in the follow-up PET scan. **(C, D)** The patient received RT with high energy electron to the right orbit.

According to the international prognostic index (IPI) scoring system, the patient was categorized as the low IPI group. Under the confirmed diagnosis of PTCL stage IIA, IFRT began after 8 cycles of CHOP (cyclophosphamide, doxorubicin, vincristine, and prednisone) regimen chemotherapy. Using VMAT providing better normal tissue sparing than 3D-CRT, the total RT dose of 50.6 Gy was delivered in 22 fractions for 4.5 weeks. After administration of 34.5 Gy, the shrinking field technique was used for the additional boost RT to the primary mass lesion (Figure 3). The patient was also assessed for salivary gland toxicity using Radiation Therapy Oncology Group (RTOG) late toxicity grading. Although the patient achieved complete remission without any morbidity relating to VMAT during the follow-up period, recurrence occurred in the orbit (including right lower eyelid) at 3 months after the treatment (Figure 4). A diagnosis of recurrence via excisional biopsy revealed PTCL, NOS. After a diagnosis of recurrence, the patient received RT with high energy electron on the right orbit (Figure 4). Eight cycles of salvage chemotherapy with IMEP (ifosfamoide, methotrexate, etoposide, and prednisolone) regimen was followed by RT. The patient died of aggravation of PTCL at 17 months after VMAT.

## Discussion

The etiology of primary BOT lymphoma is little known, although some cases of lymphomas have been reported in association with the acquired immune deficiency syndrome (Delecluse et al.^1997^). PTCLs is reported to be distinct from cutaneous T- cell lymphoma and have poor prognosis (Foss et al.^2011^). According to the fourth edition of the World Health Organization Classification of Tumors of Hematopoietic and Lymphoid Tissues (Swerdlow et al.^2008^). PTCL, NOS is classified into the nodal lymphoma group in which, along with extranodal and leukemic groups, most of aggressive T-cell lymphoma are included (Campo et al.^2011^).

Patients with PTCL had a very poor outcome compared with patients with aggressive B-cell lymphoma. The International T-cell Lymphoma Study showed that the overall survival and failure-free survival with PTCL, NOS at 10 to 15 years was 10% (Vose et al.^2008^). Suzzumiya et al. reported that in aggressive T-cell lymphomas, patients with high IPI score had adverse outcomes compared with patients with low IPI, similar to diffuse large B-cell lymphoma. However, they showed that even patients in the best IPI score group did not have a favorable survival. In this study, the patient showed a bad prognosis even with a low IPI score (Suzumiya et al.^2009^).

There have been many clinical and laboratory results, which evaluate the molecular and immunohistochemical prognostic factors in PTCL subtypes. Went et al. reported that the proliferation-associated protein Ki-67 was prognostically relevant and a new predictive score including age (>60 years), high LDH, poor performance status, and Ki-67 ≥ 80%. this score was associated with the patient outcome (Went et al.^2006^). In our study, the patient's Ki-67 was expressed with a high level (about 80%). These results seem to also affect the poor outcomes in this case. In this patient, diagnostic lumbar puncture to rule out central nervous system involvement (CNS) was not performed. In our institution, lumbar puncture is performed if the physician suspects that lymphoma has spread to the central nervous system or bone marrow. In addition, lumbar puncture is performed routinely for aggressive lymphomas (such as primary CNS lymphoma), paranasal sinus, parameningeal, testicular involvement, and patients with lymphobastic lymphoma, Burkitt lymphoma, and blastic variants.

Although there was a poor outcome in our patient, we identified the usefulness of VMAT. During the follow-up period, the patient did not develop any morbidities relating to VMAT, including xerostomia. The patient did not develop grade ≥1 xerostomia according to the RTOG criteria. Xerostomia after RT for Waldeyer's NHL was known to be a considerable morbidity and parotid-sparing RT techniques was recommended to reduce these complications (Chang et al.^2009^). Traditional RT technique for Waldeyer`s NHL is opposed lateral fields to cover the primary tumor lesion and neck lymph nodes. This RT field also includes the bilateral parotid glands. Therefore, most of patients treated with traditional RT technique experience xerostomia which is permanent toxicity and can alleviate the patient's quality of life. The most well-known parotid sparing technique, intensity modulated radiation therapy (IMRT), improves RT induced xerostomia related quality of life compared to traditional RT technique (Gupta et al.^2012^; van Rij et al.^2008^). VMAT is the next version of IMRT and more fast and efficient than IMRT (Verbakel et al.^2009^).

Although there is no consensus on the question of how to best treat primary BOT lymphoma, the recommended treatment modality for patients with early stage is combined therapy consisting of chemotherapy and RT (You et al.^2004^). T-cell lymphoma have traditionally been treated much like the B-cell lymphomas, with a combination chemotherapy regimen. Currently, there is no standard first-line regimen for the treatment of PTCL (Foss et al.^2011^). The most common chemotherapeutic regimen for the treatment of PTCL is the CHOP regimen (Savage^2011^). According to the trend described above, our patient also received CHOP regimen followed by RT. For patients with recurrent PTCL, the optimal therapeutic management is unclear, and data regarding the outcome for relapsed patients is limited (Lunning et al.^2013^). The second-line combination regimens are reported to be similar to those studied in relapsed aggressive B-cell lymphomas. These regimens are ICE (ifosphamide, carboplatin, and etoposide), DHAP (dexamethasone, cytarabine, and cisplatin), and ESHAP (etoposide, methylprednisolone, cisplatin, and cytarabine). In this study, eight cycles of salvage chemotherapy with IMEP regimen was followed by RT. Because the recurrence was localized within the orbit at the time of diagnosis of the recurrence, the patient received RT before salvage chemotherapy.

For improving the treatment outcome in primary and relapsed PTCL patients, new therapeutic agents are currently used or in clinical trials. The agents showing activity in PTCL are as follows: immunomodulatory agents (brentuximab, alemtuzumab, and lenalidomide), antifolates (pralatrexate), nucleoside analogs (gemcitabine), histone deacetylase inhibitors (belinostat, vorinostat, and romidepsin) (Petrich and Rosen^2013^; Karlin and Coiffier^2014^; Foss et al.^2011^).

Furthermore, the trials comparing autologous and allogenic hematopoietic cell transplantation in eligible patients were also initiated by the German High-Grade Non-Hodgkin Lymphoma Study Group (Shustov^2013^). The results of these trials might help elucidate difficult treatment decisions for relapsed PTCL. Some authors believe that relapsed PTCL should be considered for allogenic stem cell transplantation, if suitable (Lunning and Horwitz^2013^).

## Conclusion

Primary PTCL of BOT is an extremely uncommon disease, and all reported studies include a small number of patients. Although the patient in our report showed an local control without any morbidity relating to RT during the follow-up period, an early PTCL recurrence was detected in the orbit after the treatment. These findings support previous reports regarding PTCL adversely affecting the patient outcomes and new therapeutic regiments to best manage recurrent PTCL is warranted.

## Consent

Written informed consent was obtained from the patient's next of kin for the publication of this report and any accompanying images.

This study was reviewed and approved by the Institutional Review Board of Gachon University Gil Hospital.

## Author’ contributions

JHL and SHL drafted and reviewed the manuscript. SHL, collected clinical data and wrote the clinical part of the paper. Both authors read and approved the final manuscript.

## Authors’ original submitted files for images

Below are the links to the authors’ original submitted files for images. Authors’ original file for figure 1 Authors’ original file for figure 2 Authors’ original file for figure 3 Authors’ original file for figure 4

## References

[CR1] Campo E, Swerdlow SH, Harris NL, Pileri S, Stein H, Jaffe ES (2011). The 2008 WHO classification of lymphoid neoplasms and beyond: evolving concepts and practical applications. Blood.

[CR2] Chang DT, Amdur RJ, Pacholke H, Mendenhall NP, Morris CG, Byer GA, Olivier KR (2009). Xerostomia in long-term survivors of aggressive non-Hodgkin’s lymphoma of Waldeyer’s ring: a potential role for parotid-sparing techniques?. Am J Clin Oncol.

[CR3] Delecluse HJ, Anagnostopoulos I, Dallenbach F, Hummel M, Marafioti T, Schneider U, Huhn D, Schmidt-Westhausen A, Reichart PA, Gross U, Stein H (1997). Plasmablastic lymphomas of the oral cavity: a new entity associated with the human immunodeficiency virus infection. Blood.

[CR4] Foss FM, Zinzani PL, Vose JM, Gascoyne RD, Rosen ST, Tobinai K (2011). Peripheral T-cell lymphoma. Blood.

[CR5] Guastafierro S, Falcone U, Celentano M, Cappabianca S, Giudice A, Colella G (2008). Primary mantle-cell non-Hodgkin’s lymphoma of the tongue. Int J Hematol.

[CR6] Gupta T, Agarwal J, Jain S, Phurailatpam R, Kannan S, Ghosh-Laskar S, Murthy V, Budrukkar A, Dinshaw K, Prabhash K, Chaturvedi P, D'Cruz A (2012). Three-dimensional conformal radiotherapy (3D-CRT) versus intensity modulated radiation therapy (IMRT) in squamous cell carcinoma of the head and neck: a randomized controlled trial. Radiother Oncol.

[CR7] Holt A, Van Gestel D, Arends MP, Korevaar EW, Schuring D, Kunze-Busch MC, Louwe RJ, van Vliet-Vroegindeweij C (2013). Multi-institutional comparison of volumetric modulated arc therapy vs. intensity-modulated radiation therapy for head-and-neck cancer: a planning study. Radiat Oncol.

[CR8] Jaffe ES (2002). Lymphoid lesions of the head and neck: a model of lymphocyte homing and lymphomagenesis. Mod Pathol.

[CR9] Karlin L, Coiffier B (2014). The Changing Landscape of Peripheral T-Cell Lymphoma in the Era of Novel Therapies. Semin Hematol.

[CR10] Kemp S, Gallagher G, Kabani S, Noonan V, O'Hara C (2008). Oral non-Hodgkin’s lymphoma: review of the literature and World Health Organization classification with reference to 40 cases. Oral Surg Oral Med Oral Pathol Oral Radiol Endod.

[CR11] Lee ST, Jayalakshmi P, Raman R (1987). Non-Hodgkins lymphoma of lingual tonsil–a case report. Singapore Med J.

[CR12] Lee SJ, Suh CW, Lee Il S, Kim WS, Lee WS, Kim HJ, Choi CW, Kim JS, Shin HJ, Consortium for Improving Survival of Lymphoma (2014). Clinical characteristics, pathological distribution, and prognostic factors in non-Hodgkin lymphoma of Waldeyer’s ring: nationwide Korean study. Korean J Intern Med.

[CR13] Lunning MA, Horwitz S (2013). Treatment of peripheral T-cell lymphoma: are we data driven or driving the data?. Curr Treat Options Oncol.

[CR14] Lunning MA, Moskowitz AJ, Horwitz S (2013). Strategies for relapsed peripheral T-cell lymphoma: the tail that wags the curve. J Clin Oncol.

[CR15] Petrich AM, Rosen ST (2013). Peripheral T-cell lymphoma: new therapeutic strategies. Oncology (Williston Park).

[CR16] Savage KJ (2011). Therapies for peripheral T-cell lymphomas. Hematology Am Soc Hematol Educ Program.

[CR17] Shustov A (2013). Controversies in autologous and allogeneic hematopoietic cell transplantation in peripheral T/NK-cell lymphomas. Best Pract Res Clin Haematol.

[CR18] Suzumiya J, Ohshima K, Tamura K, Karube K, Uike N, Tobinai K, Gascoyne RD, Vose JM, Armitage JO, Weisenburger DD, International Peripheral T-Cell Lymphoma Project (2009). The International Prognostic Index predicts outcome in aggressive adult T-cell leukemia/lymphoma: analysis of 126 patients from the International Peripheral T-Cell Lymphoma Project. Ann Oncol.

[CR19] Swerdlow SH, Campo E, Harris NL, Jaffe ES, Pileri SA, Stein H, Thiele J, Vardiman JW (2008). WHO Classification of Tumours of Haematopoietic and Lymphoid Tissues. World Health Organization Classification of Tumours of Haematopoietic and Lymphoid Tissue 4th.

[CR20] Van Rij CM, Oughlane-Heemsbergen WD, Ackerstaff AH, Lamers EA, Balm AJ, Rasch CR (2008). Parotid gland sparing IMRT for head and neck cancer improves xerostomia related quality of life. Radiat Oncol.

[CR21] Verbakel WFAR, Cuijpers JP, Hoffmans D, Bieker M, Slotman BJ, Senan S (2009). Volumetric Intensity-Modulated Arc Therapy Vs. Conventional IMRT in Head-and-Neck Cancer: A Comparative Planning and Dosimetric Study. Int J Radiat Oncol Biol Phys.

[CR22] Vose J, Armitage J, Weisenburger D (2008). International peripheral T-cell and natural killer/T-cell lymphoma study: pathology findings and clinical outcomes. J Clin Oncol.

[CR23] Went P, Agostinelli C, Gallamini A, Piccaluga PP, Ascani S, Sabattini E, Bacci F, Falini B, Motta T, Paulli M, Artusi T, Piccioli M, Zinzani PL, Pileri SA (2006). Marker expression in peripheral T-cell lymphoma: a proposed clinical-pathologic prognostic score. J Clin Oncol.

[CR24] You JY, Chi KH, Yang MH, Chen CC, Ho CH, Chau WK, Hsu HC, Gau JP, Tzeng CH, Liu JH, Chen PM, Chiou TJ (2004). Radiation therapy versus chemotherapy as initial treatment for localized nasal natural killer (NK)/T-cell lymphoma: a single institute survey in Taiwan. Ann Oncol.

[CR25] Zapater E, Bagán JV, Carbonell F, Basterra J (2010). Malignant lymphoma of the head and neck. Oral Dis.

