# Supplementary figures and images for: A poor prognostic case of peripheral T-cell lymphoma in the base of tongue with chemotherapy followed by radiation therapy
Source: Springerplus. 2014 Dec 13;3:731. doi: 10.1186/2193-1801-3-731 (PMC4320182; doi:10.1186/2193-1801-3-731)

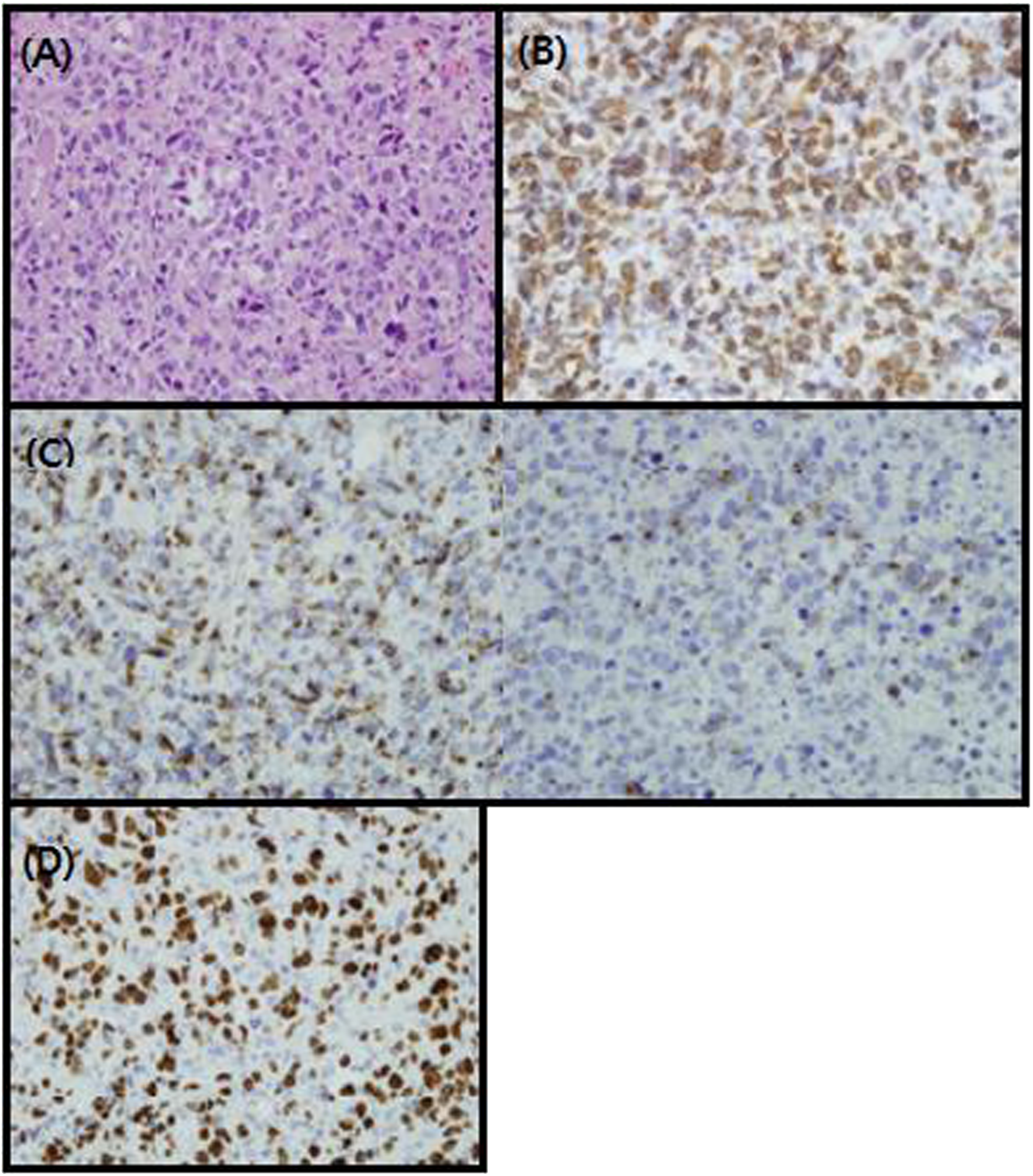

Supplement: Supplementary file 1 — Authors’ original file for figure 1 [file 40064_2014_1516_MOESM1_ESM.tif]

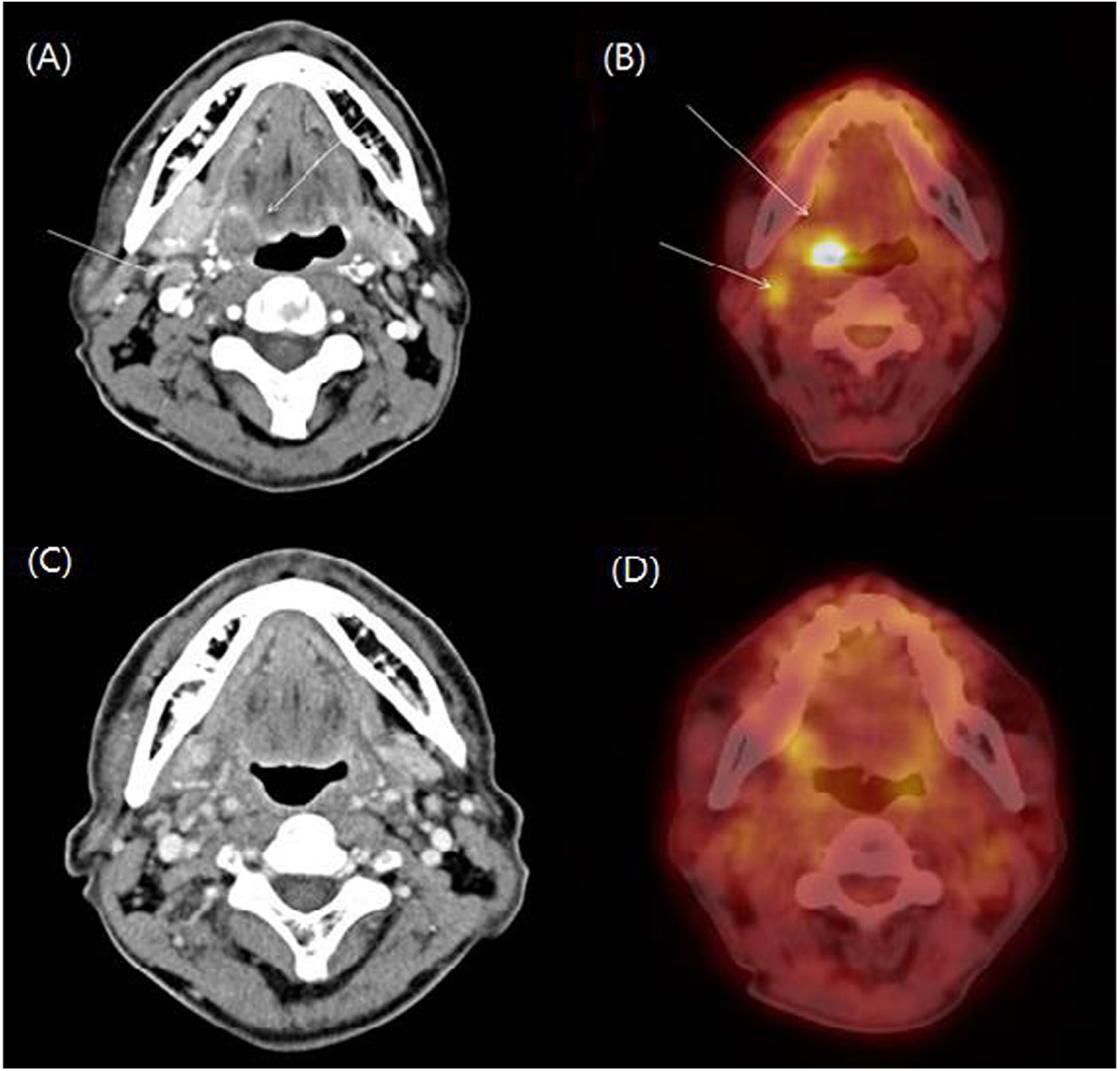

Supplement: Supplementary file 2 — Authors’ original file for figure 2 [file 40064_2014_1516_MOESM2_ESM.tif]

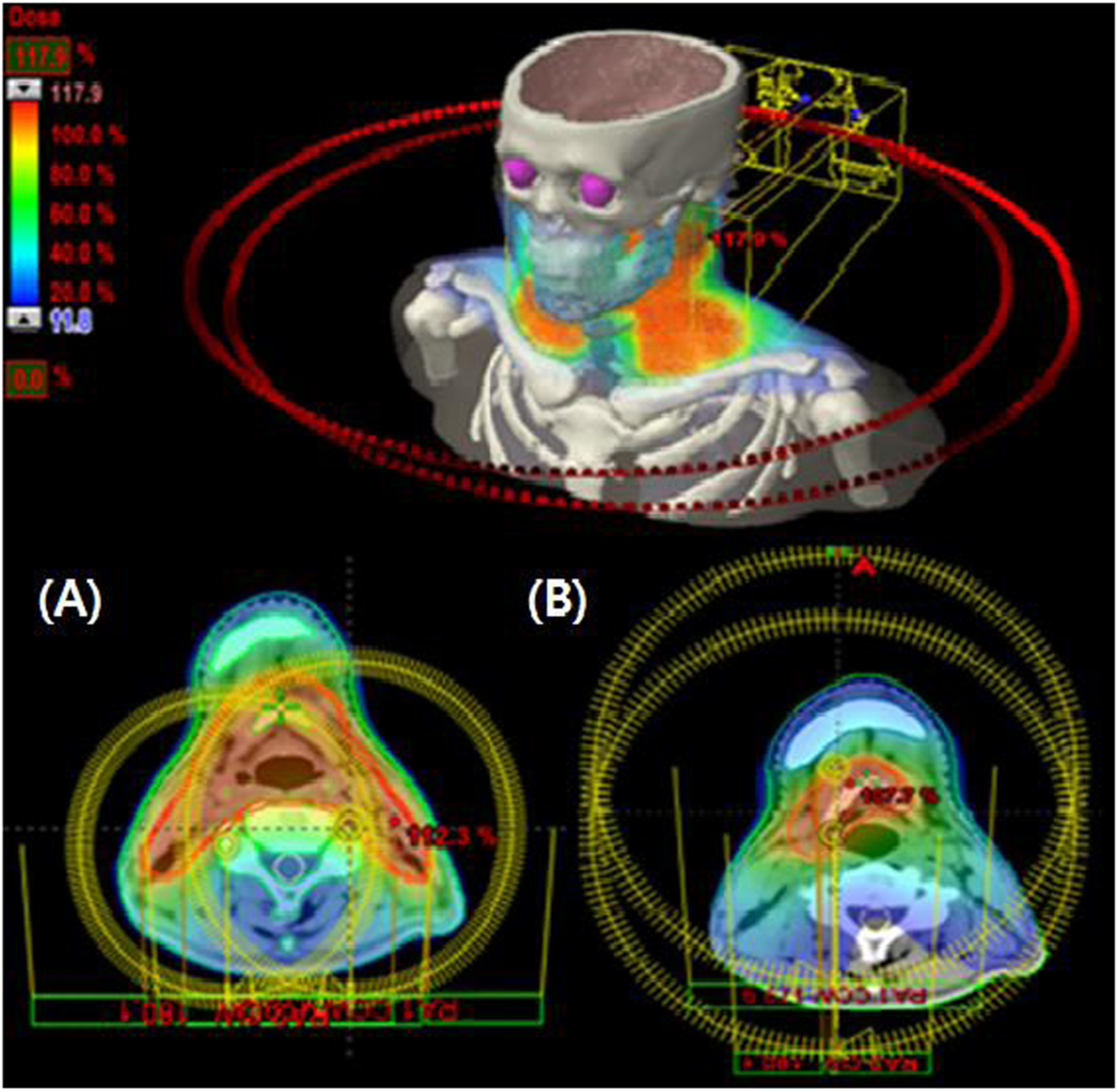

Supplement: Supplementary file 3 — Authors’ original file for figure 3 [file 40064_2014_1516_MOESM3_ESM.tif]

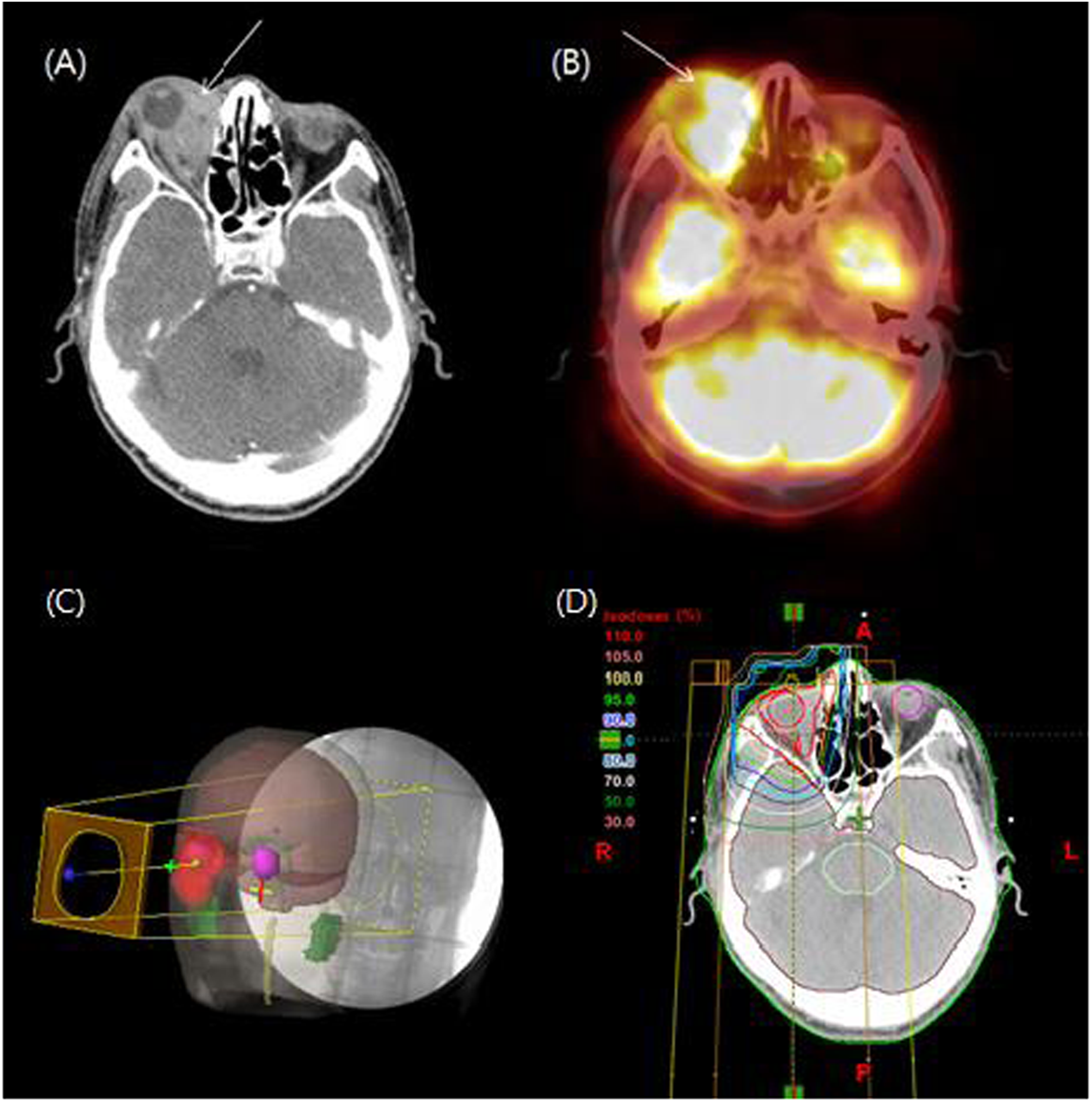

Supplement: Supplementary file 4 — Authors’ original file for figure 4 [file 40064_2014_1516_MOESM4_ESM.tif]
